# A retrospective study of the management of thoracic injury in Surabaya, Indonesia: twenty-six years experiences (1987-2012)

**DOI:** 10.1186/1749-8090-8-S1-P40

**Published:** 2013-09-11

**Authors:** PL Tahalele, Puruhito, A Prasmono, H Kusbijanto, H Soebroto, YE Sembiring

**Affiliations:** 1 Department of Surgery Division of Cardiothoracic and Vascular Surgery School of Medicine Airlangga University - Dr. Soetomo General Hospital Surabaya, Indonesia

## Background

Thoracic injuries are a major cases of mortality during the “golden hour” of trauma. Less than 10% of all blunt thoracic injuries require a emergency thoracotomy and many potentially life threathening condition can be relieved by principle procedure, such as chest tube insertion. To describe general features correlated to incidence, diagnosis, etiology, sex, age, severity and treatment of the thoracic injury patients and the way to manage it.

## Methods

The retrospective study of the 3115 thoracic injury patients treated during the period of January 1987- June 2012 (26 year) at the Emergency Department of Surgery Dr. Soetomo Hospital Surabaya provides comprehensive data concerning thoracic injury which collected and analyzed.

## Results

In the past 26 years, the total number of trauma victims treated at the Emergency Department of Surgery as 432639 patients, among whom 3473 patients were thoracic injury. They were 2949 male and 524 female. The oldest patient was 90 years old as a victim of traffic accident and the youngest was 2 month old as a victim of house accident. The numbers of blunt and penetrating trauma patients were 2973 and 500 respectively. The etiology of thoracic trauma consists of 2536 patients (73.02%) due to traffic accident; 625 patients (17.99%) due to criminality; 214 patients (6.17%) due to occupation accident; and 98 patients (2.82%) due to house accident. The majority patients were treated non-operatively (± 53.51%), drainage intra-thoracic (± 41.51%) and explorative thoracotomy (± 4.98%). There were 80 hospital deaths (2.3%), all with severe multi-trauma.

## Conclusion

The majority of the thoracic injury patient received at the department of surgery Dr.Soetomo Hospital Surabaya were treated non-operatively (± 53.51%). The overall mortality rate of thoracic injury was 2.3%

